# Assembly and phylogenetic analysis of complete chloroplast genome of a wild tobacco *Nicotiana Attenuata*

**DOI:** 10.1080/23802359.2017.1398600

**Published:** 2017-11-06

**Authors:** Haifeng Lin, Di Bai

**Affiliations:** a College of Information Science and Technology, Nanjing Forestry University, Nanjing, Jiangsu, China;; b College of Engineering, Nanjing Agricultural University, Nanjing, Jiangsu, China

**Keywords:** *Nicotiana attenuata*, chloroplast genome, wild tobacco, phylogeny

## Abstract

*Nicotiana attenuata* (*N. attenuata*) is an annual herb that induces nicotine, volatiles and phenolics in response to damage. In this study, we assembled the complete chloroplast (cp) genome of *N. attenuata* with the length of 155,886 bp, which was composed of a large single copy region (LSC) of 86,602 bp, a small copy (SSC) region of 18,518 bp and a pair of inverted repeats (IR) of 25,383 bp. This typical cp genome contains 112 unique genes, including 78 protein-coding genes, 30 tRNA genes and four rRNA genes. Phylogenetic analysis of 32 plant cp genomes based on 76 conserved protein-coding genes shows that *N. attenuata* is evolutionarily close to *N. tabacum*, then *Capsicum annuum*. The complete cp genome sequence of *N. attenuata* may provide a crucial foundation for the study of evolution, molecular biology and genetic engineering in *Nicotiana* species.

*Nicotiana attenuata* is a species of wild tobacco known locally by coyote tobacco, which is native to western North America and grows in many types of habitat. It is a glandular and sparsely hairy annual herb that can grow tall and compact or shrub-like to several feet tall and wide. *Nicotiana attenuata* can produce nicotine, a potent alkaloid that affects muscles, to master itself. In the case of *N. attenuata*, the concentration of nicotine can be up to three times the value found in common tobacco, *N. tabacum*, and it is so efficient in paralyzing or killing insects that it was widely used as an insecticide in the past (Baldwin et al. 1994).

Chloroplast is the green plastid observed in land plants, algae and some protists. The main role of chloroplasts is to conduct photosynthesis, while they also carry out lots of other functions, including fatty acid synthesis, much amino acid synthesis, and the immune response in plants. Generally, plant cp genomes are found to be highly conserved in gene order, content and organization (Henry and Henry 2005).

The leaf material from one single rosette-stage *N. attenuata* was harvested to DNA extraction in Washington County, UT (geographic coordinate: 37°16′48″N, 113°31′12″W) (Xu et al. 2017). The genomic DNA sequenced by Roche 454 and Illumina HiSeq2000 technologies was isolated with the CTAB-method (Bubner et al. 2004), and then deposited in Max Planck Institute for Chemical Ecology, 07745 Jena, Germany. The complete cp genome of *N. attenuata* was assembled using Newbler 3.0 (Wu et al. 2017), and corrected using BWA and SAMtools (Bi et al. 2016). The total length of the cp genome was 155,886 bp and then submitted to GenBank with accession number MF577082.

The complete cp genome of *N. attenuata* exhibits a typical quadripartite structure of a LSC region of 86,602 bp, a SSC region of 18,518 bp and a pair of IR region of 25,383 bp. The overall GC content of this cp genome was 37.87%, the GC content of the LSC regions was 35.97%, the IR region was 43.20%, and the SSC region was 32.06%. Using the online annotation program DOGMA (Wyman et al. 2004), a total of 129 functional genes were predicted in the *N. attenuata* cp genome, including 112 unique genes, and 17 genes were found to have two copies. These unique genes were composed of 78 protein-coding genes, 30 tRNA genes and four rRNA genes. The multi-copied genes included six protein-coding genes (*ndhB*, *rpl2*, *rpl23*, *rps12*, *rps7,* and *ycf2*), seven tRNA genes (*trnA-UGC*, *trnI-CAU*, *trnI-GAU*, *trnL-CAA*, *trnN-GUU*, *trnR-ACG,* and *trnV-GAC*), and four rRNA genes (*rrn4.5*, *rrn5*, *rrn16,* and *rrn23*). The majority of the 129 genes in the *N. attenuata* cp genome did not contain introns, whereas 18 genes contained one intron (11 protein-coding and seven tRNA genes) and two (*ycf3*, *clpP*) contained two introns. Additionally, the *rps12* gene was trans-spliced, whose exon at the 5′ end was located in the LSC region and another multi-copied exon at the 3′ end was in the IR region. In order to confirm the phylogenetic position of *N. attenuata* in plants, a total of 76 protein-coding genes from 32 plant cp genomes were selected to reconstruct the neighbour-joining phylogenetic tree using MEGA6 (Tamura et al. 2013). As illustrated in Figure 1, the phylogenetic tree strongly support that the cp genome of *N. attenuata* is evolutionarily closely related to another tobacco species *Nicotiana tabacum*, then *Capsicum annuum*.

**Figure 1.**
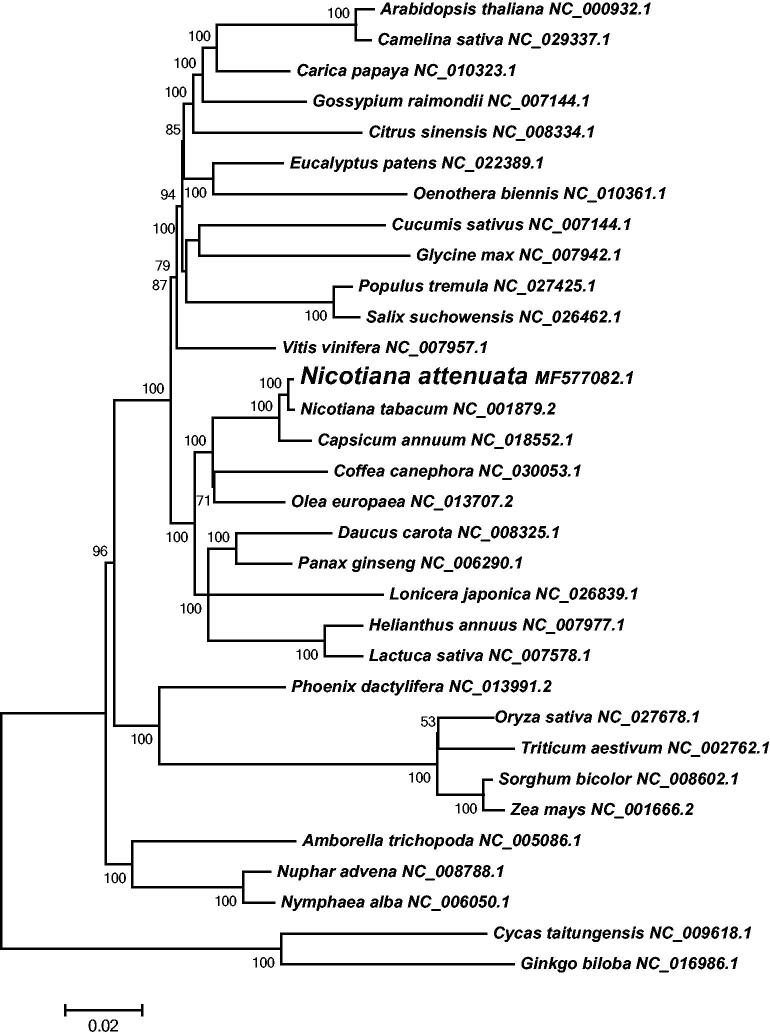
The neighbour-joining tree was constructed based on 76 protein-coding genes from 32 plant species. These protein-coding genes were aligned with MUSCLE and the phylogenetic tree was constructed using the neighbour-joining method in MEGA6. Numbers at the nodes are bootstrap support values.
